# hTERT-immortalized gingival fibroblasts respond to cytokines but fail to mimic primary cell responses to *Porphyromonas gingivalis*

**DOI:** 10.1038/s41598-021-90037-5

**Published:** 2021-05-24

**Authors:** Katarzyna B. Lagosz-Cwik, Aleksandra Wielento, Weronika Lipska, Malgorzata Kantorowicz, Dagmara Darczuk, Tomasz Kaczmarzyk, Susan Gibbs, Jan Potempa, Aleksander M. Grabiec

**Affiliations:** 1 Department of Microbiology, Faculty of Biochemistry, Biophysics and Biotechnology, Jagiellonian University, Gronostajowa 7, 30-387, Kraków, Poland; 2 Department of Periodontology and Clinical Oral Pathology, Faculty of Medicine, Jagiellonian University Medical College, Kraków, Poland; 3 Department of Oral Surgery, Faculty of Medicine, Jagiellonian University Medical College, Kraków, Poland; 4 Department of Oral Cell Biology, Academic Centre for Dentistry Amsterdam (ACTA), University of Amsterdam, Vrije Universiteit Amsterdam, Amsterdam, The Netherlands; 5 Department of Molecular Cell Biology and Immunology, Amsterdam UMC, Vrije Universiteit Amsterdam, Amsterdam, The Netherlands; 6 Department of Oral Immunology and Infectious Diseases, University of Louisville School of Dentistry, Louisville, KY USA

**Keywords:** Cellular microbiology, Pathogens, Antimicrobial responses, Inflammation

## Abstract

In periodontitis, gingival fibroblasts (GFs) interact with and respond to oral pathogens, significantly contributing to perpetuation of chronic inflammation and tissue destruction. The aim of this study was to determine the usefulness of the recently released hTERT-immortalized GF (TIGF) cell line for studies of host–pathogen interactions. We show that TIGFs are unable to upregulate expression and production of interleukin (IL)-6, IL-8 and prostaglandin E2 upon infection with *Porphyromonas gingivalis* despite being susceptible to adhesion and invasion by this oral pathogen. In contrast, induction of inflammatory mediators in TNFα- or IL-1β-stimulated TIGFs is comparable to that observed in primary GFs. The inability of TIGFs to respond directly to *P. gingivalis* is caused by a specific defect in Toll-like receptor-2 (TLR2) expression, which is likely driven by *TLR2* promoter hypermethylation. Consistently, TIGFs fail to upregulate inflammatory genes in response to the TLR2 agonists Pam2CSK4 and Pam3CSK4. These results identify important limitations of using TIGFs to study GF interaction with oral pathogens, though these cells may be useful for studies of TLR2-independent processes. Our observations also emphasize the importance of direct comparisons between immortalized and primary cells prior to using cell lines as models in studies of any biological processes.

## Introduction

Fibroblasts are a heterogeneous population of resident stromal cells of mesenchymal origin that maintain tissue homeostasis mainly by production and turnover of extracellular matrix^1,2^. It is now recognized that they are also important sentinel cells that express several classes of pattern recognition receptors^2^. This means that they can respond to pathogenic microorganisms and danger signals by producing a broad range of inflammatory mediators^3^. Fibroblasts may also drive the persistent inflammation that underlies several pathologies and can lead to tissue damage^4^. Therefore, there is a growing need for a better understanding of fibroblast responses in pathologies affecting connective tissues that are caused by non-resolving inflammation.

Among inflammatory diseases that directly or indirectly affect connective tissues, periodontitis is the most common pathology, with its severe form affecting more than 10% of the population^5^. In periodontitis, chronic inflammation is initiated and sustained by the dysbiotic oral microflora that drives and sustains excessive activation of infiltrating immune cells and resident cells within the gingival tissue^6^. While the majority of research on host–pathogen interactions in periodontitis has focused on gingival epithelial cells (GECs), which are in direct contact with the dental plaque, it is now appreciated that gingival fibroblasts (GFs) also interact with, and potently respond to, oral pathogens that penetrate the epithelial barrier^3^. Given the abundance of GFs in inflamed gingival tissue, they significantly contribute to the perpetuation of chronic inflammation in periodontitis.

Progress in understanding GF biology has been hampered by the short life span of primary cells in culture. GFs quickly undergo senescence, which is associated with reduced proliferation, elevated expression of cell cycle inhibitors, cytoskeletal changes, impairment of extracellular matrix (ECM) production and defects in the organization of ECM fibres^7,8^. This, in combination with high variability between primary cell lines derived from different donors^9^, has stimulated interest in the generation of immortalized GF lines that mirror primary cell responses. Overexpression of human telomerase reverse transcriptase (hTERT) is a commonly used strategy for immortalization of human cells^10^. hTERT maintains the length of telomeres, which in many cell types is sufficient for immortalization without causing cancer-associated changes or altering phenotypic properties^11^. Due to genetic stability and phenotypic similarities with primary cells, hTERT-immortalized cell lines are commonly used in basic research and in tissue engineering^12^.

Due to the appealing properties of a longer life-span and no donor variability, the recently released hTERT-immortalized GF (TIGF) cell line has been widely used in basic and translational studies. TIGFs have been successfully introduced as a cytotoxicity model in studies of dental materials^13,14^, as components of organotypic 3-dimensional gingival tissue models^15–17^, and as a model cell line in molecular research utilizing the CRISPR/Cas9 genome editing technology^18^. However, it has not been tested to what extent TIGFs mimic inflammatory responses of primary human GFs (PHGFs) upon interaction with oral pathogens. Here, we show that TIGFs are susceptible to adhesion and invasion by the oral pathogen *Porphyromonas gingivalis* and that their responses to cytokine stimulation are comparable to those observed in primary GFs. However, they fail to upregulate inflammatory mediators in response to infection with *P. gingivalis* and Toll-like receptor-2 (TLR2) agonists. This is likely due to an epigenetic defect in TLR2 promoter methylation that may be responsible for an absence of TLR2 protein expression in TIGFs.

## Results

### TIGFs internalize *P. gingivalis* but fail to upregulate inflammatory mediators after bacterial challenge

**Figure 1:**
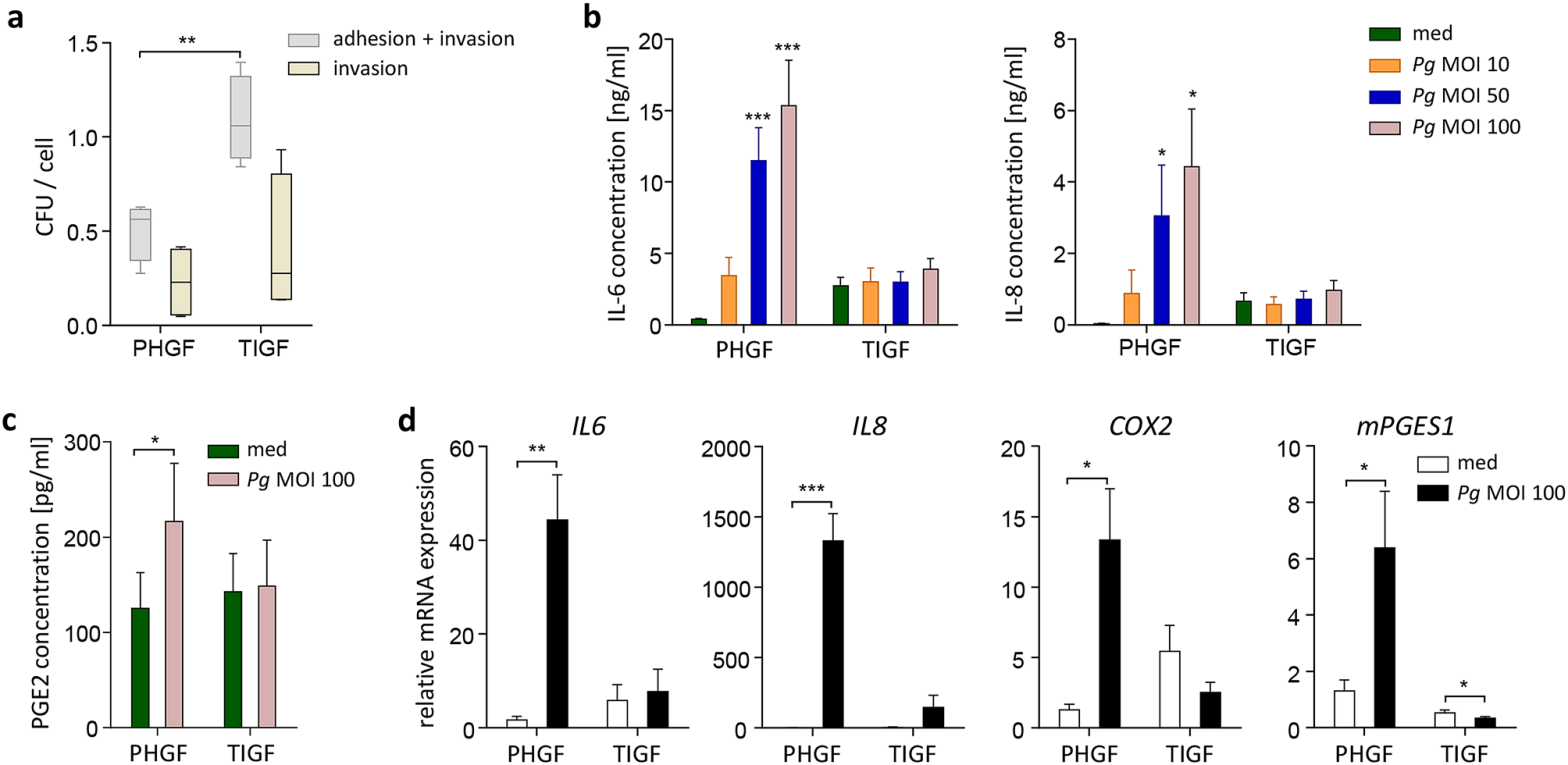
TIGFs internalize *P. gingivalis* but fail to mount an inflammatory response after bacterial challenge. (**a**) *P. gingivalis* internalization by PHGFs and TIGFs determined by colony-forming assay. Cells were infected with *P. gingivalis* (MOI 100) for 1 h and then cultured for another 1 h in medium, with or without antibiotics. Results of four independent experiments are presented as boxplots, where the line within the box denotes the median number of colony-forming units (CFU)/cell, the boxes represent the 25th and 75th percentiles, and the lines outside the box mark the minimum and maximum values. **P < 0.01; unpaired t-test (**b**) Secretion of IL-6 and IL-8 by PHGFs and TIGFs (n = 6–7) infected with increasing MOI of *P. gingivalis* (10, 50, 100) for 1 h followed by washing, then 23 h culture in fresh medium. *P < 0.05; ***P < 0.001; 1-way ANOVA followed by Bonferroni multiple comparison test. (**c**) Production of PGE2 by PHGFs and TIGFs (n = 8–9) infected with *P. gingivalis* (MOI 100) for 24 h. *P < 0.05; paired t-test. Data in (**b,c**) are presented as mean concentration + SEM. (**d**) Relative mRNA expression of *IL6*, *IL8*, *COX2* and *mPGES1* in PHGFs and TIGFs (n = 5–7) infected with *P. gingivalis* (MOI 100) for 24 h. Data are presented as mean relative expression + SEM. *P < 0.05; **P < 0.01; ***P < 0.001; paired t-test.

To verify whether TIGFs provide a useful tool for studies of interactions between oral pathogens and host cells, we compared adhesion and invasion of *P. gingivalis* in TIGFs and primary cells using the colony-forming assay. Significantly more bacteria adhered to the surface of TIGFs compared to PHGFs, and comparable numbers of live intracellular bacteria were detected in both cell types (Fig. 1a), indicating that TIGFs retain the ability to internalize bacteria. Next, we compared the effects of bacterial challenge on PHGF and TIGF inflammatory activation. Infection with increasing multiplicity of infection (MOI) of *P. gingivalis* resulted in a dose-dependent increase in IL-6 and IL-8 production by PHGFs (Fig. 1b). However, the presence of bacteria had little impact on cytokine production by TIGFs, shown by only a slight increase in IL-6 and IL-8 levels in cells infected with the highest tested *P. gingivalis* MOI, compared to uninfected cells (Fig. 1b). *P. gingivalis* also induced a two-fold increase in prostaglandin E2 (PGE2) secretion in PHGFs, whereas in TIGFs, PGE2 production remained unaffected (Fig. 1c). Consistently, mRNA expression of *IL6*, *IL8*, *COX2,* and *mPGES1* was significantly upregulated in PHGFs, but not in TIGFs following infection (Fig. 1d). Of note, basal cytokine production as well as expression of *IL6*, *IL8,* and *COX2* was evidently higher in TIGFs than in PHGFs (Fig. 1b,d). Taken together, these data showed that TIGFs are unable to mount an inflammatory response to *P. gingivalis*, despite their ability to secrete large amounts of cytokines under homeostatic conditions and to internalize bacteria.

### TNFα and IL-1β induce comparable levels of inflammatory mediators in TIGFs and PHGFs

**Figure 2:**
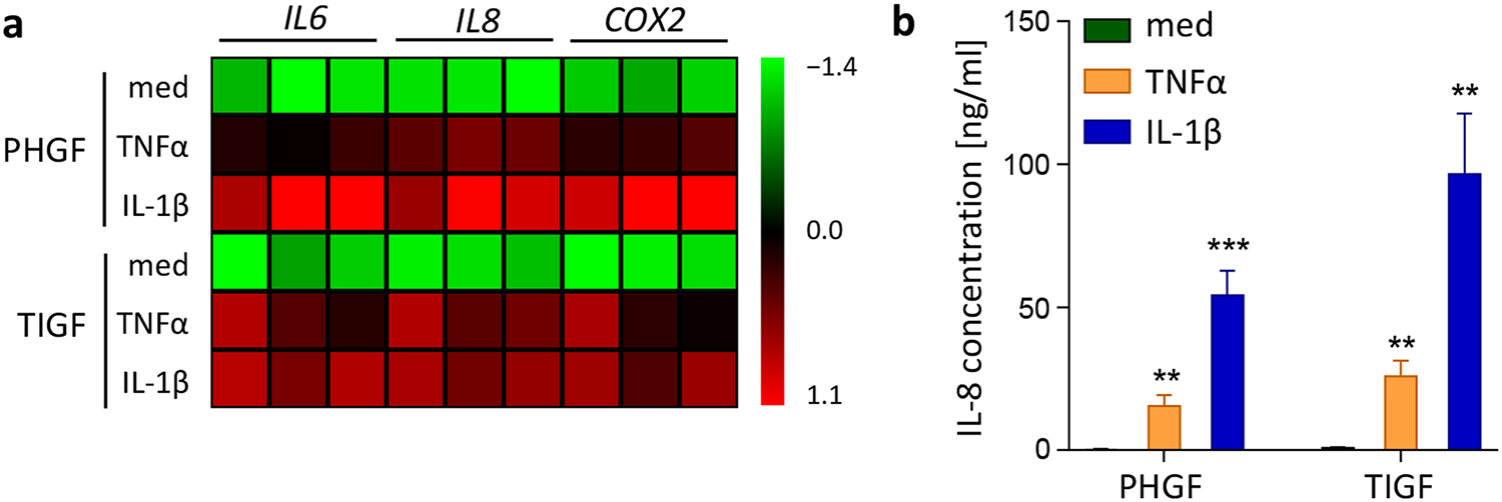
TIGFs and PHGFs respond to cytokine stimulation in a similar manner. (**a**) Relative mRNA expression of *IL6*, *IL8* and *COX2* in PHGFs and TIGFs that were left unstimulated in medium (med) or were stimulated with TNFα (10 ng/ml) or IL-1β (10 ng/ml) for 24 h. Data for PHGFs from individual donors (n = 3) and TIGFs from independent experiments (n = 3) are shown on a heat map as column Z-scores calculated from ΔCt values relative to a housekeeping gene (*RPLP0*). (**b**) Secretion of IL-8 by PHGFs and TIGFs (n = 8) and stimulated as in (**a**). Data are presented as mean concentration + SEM. **P < 0.01; ***P < 0.001; 1-way ANOVA followed by Bonferroni multiple comparison test.

Next, we tested whether the inability of TIGFs to upregulate cytokine and *COX2* expression is specific for bacterial challenge, or whether it represents a global defect in inflammatory transcriptional programs. To verify this, we stimulated PHGFs and TIGFs with cytokines that play a central role in the pathogenesis of periodontitis and are present in large quantities in the inflamed gingival tissue^19^. TNFα and IL-1β stimulation potently induced *IL6, IL8,* and *COX2* expression in both cell types, though at 4 h post stimulation gene induction by IL-1β was more pronounced in PHGFs than in TIGFs (Fig. 2a). Cytokine-induced IL-8 production by both cell types was also comparable (Fig. 2b), suggesting that TIGFs are capable of eliciting an inflammatory response.

### Cytokines, but not *P. gingivalis*, stimulate NF-κB and MAP kinase signaling in TIGFs

**Figure 3:**
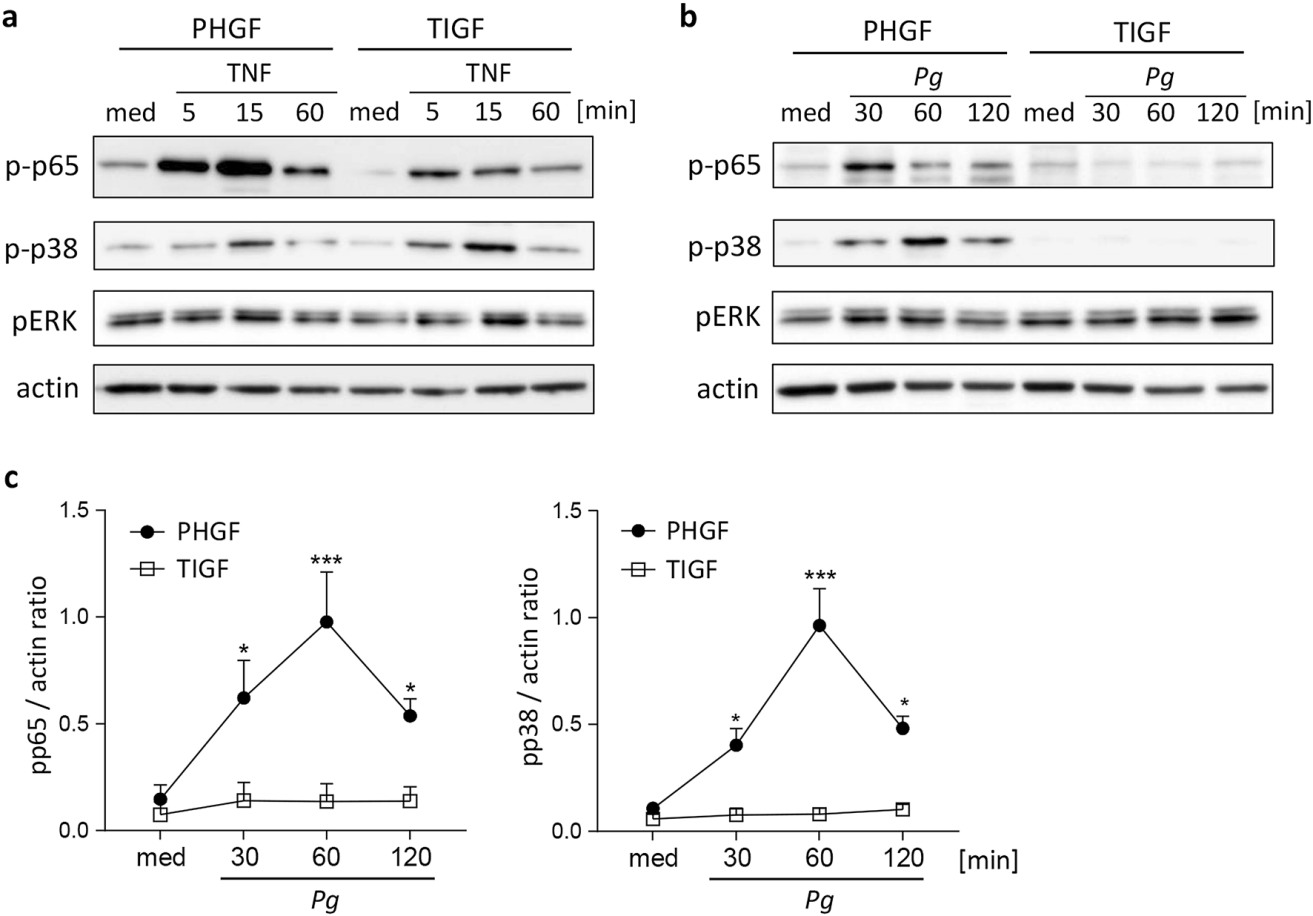
TNFα stimulation, but not *P. gingivalis* infection, activates NF-κB and MAP kinase signaling in TIGFs. Western blot analysis of p65, p38, and ERK phosphorylation in PHGFs and TIGFs (**a**) after stimulation with TNFα (10 ng/ml) or (**b**) upon infection with *P. gingivalis* (MOI 50) for the indicated time points. Actin was used as loading control and medium (med) indicates unstimulated/uninfected cells. Results shown are representative of 3–5 independent experiments and full-length blots are presented in Supplementary Fig. S1. (**c**) Densitometric analysis of p38 and p65 phosphorylation in *P. gingivalis*-infected cells (n = 4–5). Data are shown as mean signal intensity relative to actin. *P < 0.05; ***P < 0.001; 1-way ANOVA followed by Bonferroni multiple comparison test.

Engagement of pattern recognition and cytokine receptors converge on the activation of NF-κB and mitogen-activated protein kinase (MAPK) signaling pathways^20^. To identify the molecular mechanism underlying the inability of TIGF to upregulate inflammatory gene transcription upon *P. gingivalis* infection, we analyzed the activation status of the components of these pathways known to be activated by cytokines and by oral pathogens: the p65 subunit of NF-κB, extracellular signal-regulated kinase (ERK), and p38 MAPK^21–23^. As expected, TNFα stimulation induced rapid phosphorylation of p38 and the p65 subunit of NF-κB, though some differences in the activation kinetics and signal intensity have been noted (Fig. 3a). In contrast, infection with *P. gingivalis* resulted in significant activation of both signaling molecules only in PHGFs, but not in TIGFs (Fig. 3b,c). ERK displayed high basal phosphorylation levels that remained largely unaffected by TNFα stimulation or *P. gingivalis* infection in both cell types (Fig. 3a,b). This suggests that alterations in early signaling events or bacterial recognition are responsible for the observed defect in TIGF inflammatory activation.

### TIGFs lack TLR2 expression

**Figure 4:**
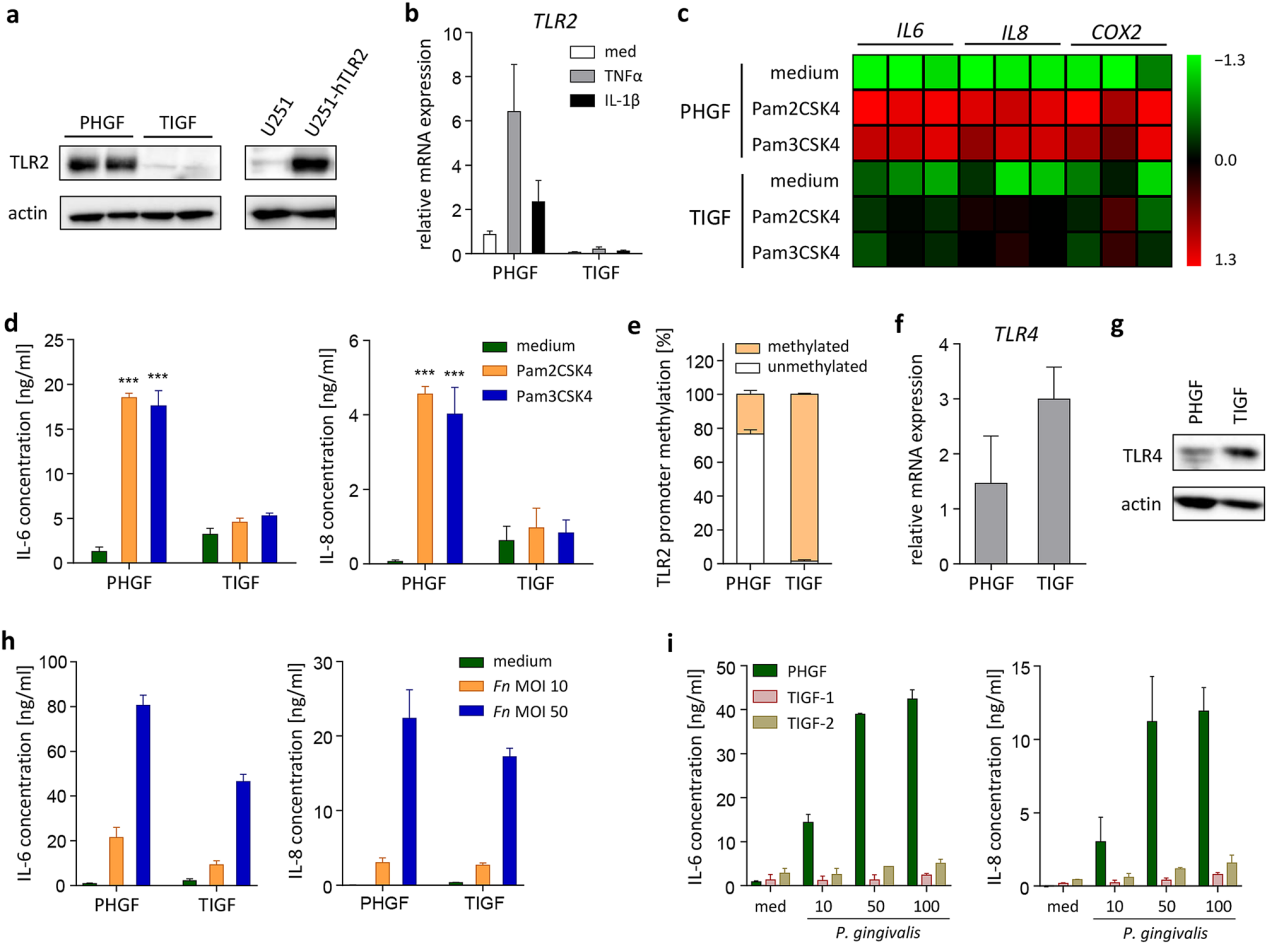
TIGFs lack TLR2 expression and fail to respond to TLR2 agonists. (**a**) Western blot analysis of TLR2 protein expression in PHGF and TIGF total cell lysates. U-251 MG cells overexpressing hTLR2 were used as a positive control and actin was used as a loading control. Results presented are representative of two independent experiments and full-length blots are presented in Supplementary Fig. S2. (**b**) Relative mRNA expression of TLR2 in PHGFs and TIGFs (n = 3) that were left unstimulated (med) or were stimulated with TNFα (10 ng/ml) or IL-1β (10 ng/ml) for 24 h. (**c**) Relative mRNA expression of *IL6*, *IL8* and *COX2* in PHGFs and TIGFs stimulated with Pam2CSK4 (1 μg/ml) or Pam3CSK4 (1 μg/ml) for 24 h. Data for PHGFs from individual donors (n = 3) and TIGFs from independent experiments (n = 3) are shown on a heat map as column Z-scores calculated from ΔCt values relative to a housekeeping gene (*RPLP0*). (**d**) Secretion of IL-6 and IL-8 by PHGFs and TIGFs (n = 3) and stimulated as in (**c**). (**e**) TLR2 promoter methylation in PHGFs and TIGFs (n = 3) analyzed using the EpiTect methyl II PCR assay and presented as mean percentage of methylated DNA + SEM. (**f**) Relative TLR4 mRNA expression in PHGFs and TIGFs (n = 3). (**g**) Western blot analysis of TLR4 protein expression in total cell lysates of PHGFs and TIGFs. Actin was used as a loading control. (**h**) Secretion of IL-6 and IL-8 by PHGFs and TIGFs (n = 3–4) infected with increasing MOI of *F. nucleatum* (10, 50) for 24 h. (**i**) Secretion of IL-6 and IL-8 by PHGFs and two independent batches of TIGFs (TIGF-1 and TIGF-2) (n = 2–3) infected with increasing MOI of *P. gingivalis* (10, 50, 100). Data are shown as mean relative mRNA expression + SEM (**b**,**f**) or mean concentration + SEM (**d**,**h**,**i**). ***P < 0.001; 1-way ANOVA followed by Bonferroni multiple comparison test.

The innate immune response to *P. gingivalis* is induced by pattern recognition receptors, among which TLR2 plays the most prominent role^24^. We hypothesized that alternations in TIGF responses to *P. gingivalis* may be caused by *TLR2* deficiency. First, we compared constitutive TLR2 protein levels in PHGFs and TIGFs using the U-251 MG cell line overexpressing human TLR2 as a positive control. Western blot analysis showed that TLR2 is readily detectable in PHGFs but is almost completely absent in TIGFs (Fig. 4a). This difference was also evident at the mRNA level and, in contrast to primary cells, the *TLR2* transcript was not inducible by TNFα or IL-1β stimulation in TIGFs (Fig. 4b). To verify the functional consequences of diminished TLR2 expression, we analyzed the effects of TLR2-specific ligands, Pam2CSK4 and Pam3CSK4, on TIGF inflammatory activation. Both ligands significantly upregulated the expression of *IL6, IL8,* and *COX2* in PHGFs, while their effects on TIGF activation were negligible (Fig. 4c). In line with mRNA data, Pam2CSK4 and Pam3CSK4 induced secretion of IL-6 and IL-8 only in primary cells, but not in TIGFs (Fig. 4d).

Epigenetic changes in DNA methylation play an important role in regulation of TLR2 expression^25^. To verify whether the observed reduction in TLR2 levels is mediated by epigenetic alterations, we compared *TLR2* promoter methylation in PHGFs and TIGFs using the EpiTect methyl II PCR assay. We found that the *TLR2* promoter region is almost completely methylated in TIGFs whereas it remains mostly unmethylated in PHGFs (Fig. 4e). This observation may explain the absence of TLR2 expression in resting TIGFs and the inability of cytokines to upregulate *TLR2* in TIGFs despite intact inflammatory signaling cascades. Notably, this defect is specific for *TLR2* as we did not observe a similar reduction in expression of *TLR4* (Fig. 4f) or NOD-like receptors NOD1 and NOD2 (Supplementary Fig. S3). In fact, we noted a trend towards elevated *TLR4* mRNA and protein expression in TIGFs compared to PHGFs, though this was not statistically significant (Fig. 4f,g). To confirm that TLR4 expressed in TIGFs is functional, we infected cells with *Fusobacterium nucleatum*. In contrast to *P. gingivalis*, which is recognized by host cells predominantly through TLR2^26,27^, *F. nucleatum* activates cells through both TLR4 and TLR2^28,29^. TIGF infection with *F. nucleatum* induced IL-6 and IL-8 secretion in TIGFs, albeit to a lesser extent than in primary cells (Fig. 4h), indicating that TIGFs are capable of responding to infection through TLR4, but this response is diminished compared to primary cells in the absence of TLR2. Finally, to exclude the possibility that the defect in TLR2 expression is specific for the batch of cells used in our experiments, we compared two independent batches of TIGFs that are used in different laboratories and found no significant differences between their inflammatory responses. Both TIGF batches (TIGF-1 and TIGF-2) were unable to upregulate IL-6 and IL-8 production upon *P. gingivalis* infection (Fig. 4i). Collectively, these results indicate that the observed defects in TIGF responses to *P. gingivalis* challenge can be attributed to diminished expression of *TLR2* and the resulting inability of TIGFs to respond to TLR2-activating signals.

## Discussion

Human cells immortalized through the overexpression of hTERT represent invaluable tools in cell biology research. A plethora of cell types from various organs and tissues have been immortalized using this method, including foreskin keratinocytes^30^, dermal papilla cells^31^, intestinal epithelial cells^32^, urothelial cells^33^, and liver endothelial cells^34^. Importantly, these cells maintain the morphology, expression profiles of cell type-specific markers, and functional characteristics of primary cells. Cells of the tooth-supporting tissues have also been successfully immortalized by hTERT overexpression. hTERT-immortalized human dental papilla cells retain the undifferentiated state of primary cells and represent a valuable model for odontoblast differentiation^35^. Similarly, the recently generated hTERT-immortalized human gingival keratinocyte (TIGK) cell line largely recapitulates the phenotypic properties and inflammatory responses of primary GECs, therefore, TIGKs are widely used for analyses of interactions between host gingival cells and oral bacteria^17,36–38^.

Here, we identify both strengths and limitations of TIGFs as a representation of primary cells for studies of GF activation upon interaction with oral pathogens and the inflammatory tissue environment. Comparing the immortalized cell line to primary GFs derived from ten healthy individuals, we reveal that TIGFs have a similar activation profile in response to stimulation with TNFα and IL-1β. In contrast, the inflammatory responses of TIGFs to oral pathogens are defective, despite similar levels of *P. gingivalis* adhesion and invasion, compared to primary GFs. This is likely due to diminished TLR2 expression which is essential for host responses to *P. gingivalis*^26,27^, but is only partially involved in cell activation by *F. nucleatum*^28,29^. Consistently, while inflammatory mediator induction by *P. gingivalis* is almost completely absent, TIGFs partly maintain the ability to respond to *F. nucleatum.* Importantly, expression of TLR4 does not compensate for the absence of TLR2 in TIGFs despite the reported ability of *P. gingivalis* LPS to activate TLR4^39^. This is not surprising in light of in vitro and in vivo studies demonstrating that host inflammatory responses to live *P. gingivalis* rely predominantly on TLR2, but not TLR4 activation^26,27,40^.

We show that TIGFs lose some phenotypic features of primary cells, while retaining others, despite their genomic stability and diploid status confirmed during extended culture^14^. The defect in TIGF activation upon infection with *P. gingivalis* observed in this study is accompanied by our previously reported intact response to gingivitis and cariogenic biofilms in organotypic culture^16^. In the latter experiments, biofilm was applied topically to reconstructed epithelium grown on a TIGF-populated hydrogel^16^. Such a method of exposure would result in release of GEC-derived cytokines, including IL-6 and IL-8, which would in turn stimulate inflammatory cytokine release from fibroblasts^41^. This would explain the apparently contradictory findings since the current study describes a direct TIGF exposure whereas an indirect exposure method was used in the previous work^16^. TIGFs also display a pattern of chemokine receptor expression similar to that observed in primary GFs^42^. Similarly, hTERT-immortalized airway smooth muscle (ASM) cells mirror the behavior of primary cells in their proliferative capacity and inflammatory responses, but their production of the ECM components fibronectin and fibulin-1 is defective^43^. Consistently, the comparison of immortalized cell lines derived from healthy donors and patients with asthma revealed that they retain some of the unique characteristics of asthmatic cells, such as hyperproliferation and excessive release of inflammatory mediators, but not the differences in ECM deposition that are seen in primary ASM cells^43^. These observations highlight the limitations of hTERT-immortalized cell lines, which tend to lose certain unique phenotypic properties of parent cells, rendering these systems not reliable for some functional studies. These specific defects can be identified only through direct, side-by-side comparisons with primary cells.

Our data indicate that the lack of TLR2 expression in TIGFs may be driven by a specific epigenetic defect in DNA methylation. Cell immortalization may promote changes in DNA methylation profiles that alter cellular functions^44^. For example, hTERT-mediated immortalization of human foreskin keratinocytes and adenoid epithelial cells causes repression of the cell cycle inhibitor p16^INK4a^ which is driven by increased promoter methylation and can be reverted by treatment with DNA methyltransferase inhibitors^45^. Similarly, hypermethylation of genes involved in DNA damage repair, apoptosis, hormone responses, and tumor invasion has been observed in a range of in vitro immortalized prostate epithelial cell lines^46^. However, relatively few changes have been noted in hTERT-immortalized cells compared to cell lines generated using different immortalization methods^46^. Genome-wide profiling of DNA methylation in human bronchial epithelial cells immortalized through the introduction of hTERT and SV40 LT revealed global genomic hypomethylation, as well as site-specific changes in DNA methylation patterns that affect genes involved in cell proliferation^47^. Interestingly, many of the epigenetic defects found in immortalized cell lines mirror those found in spontaneously transformed cells and cancer tissues^46,47^. It is well-established that expression of TLR2 is dynamically regulated by epigenetic mechanisms. Increased methylation of the TLR2 promoter region has been reported in periodontitis^48^ and is induced in GECs by chronic exposure to oral pathogens^25^. It is therefore not surprising that TLR2 expression is altered in a cell line that has dysregulated epigenetic regulatory mechanisms. Curiously, TLR4 expression was not affected in TIGFs compared to primary cells, suggesting that epigenetic regulation of TLR2 and TLR4 is likely driven by independent mechanisms. Consistently, distinct TLR2 and TLR4 promoter methylation patterns have been reported in periodontitis and Behçet's disease^49,50^. While our results identify an aberrant promoter methylation profile of a single gene, the full scope of possible alterations in DNA methylation in TIGFs remains to be determined.

Collectively, the results presented here show important limitations of using TIGFs to evaluate GF behavior under inflammatory conditions, and provide an independent confirmation that TLR2 is indispensable for GF activation in response to infection with *P. gingivalis*. Nonetheless, given their genetic stability and intact signal transduction pathways that drive inflammatory gene transcription, TIGFs may be a useful model for studies of TLR2-independent processes. Finally, our data also highlight the notion that the study of any biological process in an immortalized cell line must first be preceded by a direct comparison with parent cells to confirm the similarity of cell responses under the tested experimental conditions.

## Materials and methods

### Cell culture

PHGFs were isolated from gingival biopsies collected from 10 healthy subjects presenting for orthodontic treatment at the Department of Periodontology and Clinical Oral Pathology, Faculty of Medicine, Jagiellonian University Medical College, Kraków, Poland. The study was approved by the Bioethical Committee of the Jagiellonian University in Krakow, Poland (approval numbers 122.6120.337.2016 and 1072.6120.104.2019) and all methods were performed in accordance with the regulations and guidelines of the Committee. Written informed consent was obtained from all donors. PHGFs were isolated as described previously^38^ and used for experiments between passage numbers 4 and 9. TIGFs (T0026) were purchased from Applied Biological Materials Inc. (Richmond, BC, Canada). Both cell types and the U-251 MG cell line were cultured in DMEM (Lonza) supplemented with 10% fetal bovine serum (FBS, Biowest), penicilin/streptomycin (50 U/ml) (Gibco) and gentamicin (50 U/ml) (Biowest). One day prior to experiments, cells were seeded in medium supplemented with 2% FBS, without antibiotics.

### Bacterial culture, cell infection and treatment with cytokines or TLR2 ligands

Wild-type *P. gingivalis* (strain ATCC33277) and *F. nucleatum* (strain ATCC10953) were grown anaerobically for 7 days on blood agar plates (brain–heart infusion [BHI, Becton–Dickinson] with yeast extract containing 0.5 mg/mL L-cysteine, 10 µg/mL hemin and 0.5 µg/mL vitamin K). Bacterial suspensions at optical density (OD)_600_ = 1 (corresponding to 10^9^ colony-forming units/ml) were prepared for cell infections as described before^51^. Figure legends indicate the multiplicity of infection (MOI) and infection times used for individual experiments.

### Colony-forming assay

Cells were seeded in duplicate wells in 12-well plates (1.25 × 10^5^ cells per well) and infected with *P. gingivalis* (MOI 100) for 1 h, then washed 3 times with PBS. Cells were then cultured either in fresh in medium without antibiotics, or in medium containing gentamicin (2.5 mg/ml) and metronidazole (2 mg/ml) for 1 h, to discriminate between adherent and internalized bacteria. Cells were subsequently lysed in distilled, sterile H_2_O for 40 min. Cell lysates were serially diluted and 10 µl of each dilution was plated on BHI blood agar plates in triplicate. Plates were incubated for 5 days at 37 °C in anaerobic conditions, then bacterial colonies were counted.

### ELISA

PHGFs and TIGFs cultured in 96-well plates (2.5 × 10^4^ cells per well) were infected with *P. gingivalis* (MOI 10, 50, or 100) or *F. nucleatum* (MOI 10 or 50) for 24 h or 1 h, washed 3 times with PBS, then cultured in fresh medium containing gentamycin (2.5 mg/ml) and metronidazole (2 mg/ml) for 23 h prior to supernatant collection. Alternatively, cells were stimulated with cytokines (10 ng/ml IL-1β or 10 ng/ml TNFα, both from BioLegend) or TLR2 ligands (1 μg/ml Pam2CSK4 or 1 μg/ml Pam3CSK4, both from Invivogen) for 24 h. IL-8 and IL-6 concentrations were determined in cell-free supernatants using ELISA MAX Standard sets (BioLegend), while PGE2 levels were measured using a Prostaglandin E2 ELISA Kit-Monoclonal (Cayman Chemical), according to the manufacturer’s instructions. A FlexStation3 Multi-Mode microplate reader (Molecular Devices) was used for absorbance measurements.

### RNA isolation and quantitative polymerase chain reaction (qPCR)

PHGFs and TIGFs cultured in 12-well plates (2.5 × 10^5^ cells per well) were either stimulated with cytokines or TLR2 ligands, or infected with *P. gingivalis* (MOI 100) for 24 h. Total RNA was extracted using an ExtractMe Total RNA isolation kit (Blirt) and quantified using a BioPhotometer D30 (Eppendorf). RNA was reverse transcribed using a High-Capacity cDNA Reverse Transcription Kit (Applied Biosystems). PowerUp SYBR Green PCR mix (Applied Biosystems) was used for qPCR reactions that were performed on a CFX96 Touch™ Real-Time PCR Detection System (Bio-Rad) and the obtained results were analyzed using the CFX Manager (Bio-Rad). mRNA expression relative to housekeeping genes (*ACTB* or *RPLP0*) was calculated using the ΔΔCT method, unless otherwise indicated. Sequences of the primers (purchased from Genomed S.A., Poland) used are listed in Table 1.

**Table 1:** Sequences of primers used for qPCR analyses.

Gene	Forward primer	Reverse primer
*IL6*	GACAGCCACTCACCTCTTCA	CCTCTTTGCTGCTTTCACAC
*IL8*	GCTCTGTGTGAAGGTGCAGT	CCAGACAGAGCTCTCTTCCA
*IL1B*	ACAGATGAAGTGCTCCTTCCA	GTCGGAGATTCGTAGCTGGAT
*COX2*	AGCCCTTCCTCCTGTGCCT	AATCAGGAAGCTGCTTTTTACCT
*mPGES1*	CACGCTGCTGGTCATCAAGAT	CCGTGTCTCAGGGCATCCT
*TLR2*	CGGAATGTCACAGGACAGCA	TACCACAGGCCATGGAAACG
*TLR4*	ACCATCATTGGTGTGTCGGTC	AGCCAGCAAGAAGCATCAGG
*NOD1*	ACAGCCAGGGCGAGATAC	AAAGGTGCTAAGCGAAGAG
*NOD2*	GATTGGCTGCCTTCCTTCTA	GAGCGTCTCTGCTCCATCAT
*RPLP0*	GCGTCCTCGTGGAAGTGACATCG	TCAGGGATTGCCACGCAGGG
*ACTB*	CCACACTGTGCCCATCTACG	AGGATCTTCATGAGGTAGTCAGTCAG

### Immunoblotting

Cells cultured in 12-well plates (2.5 × 10^5^ cells per well) were lysed in Laemmli’s buffer containing 2% SDS, 10% glycerol and 125 mM Tris–HCl, pH 6.8. Protein concentration was measured using the Bradford assay (BioShop) and equal amounts of protein were resolved by electrophoresis on 10% polyacrylamide gels. Proteins were then transferred to PVDF membranes using a Trans-Blot Turbo Transfer System (Bio-Rad). The membranes were then blocked using 5% milk (BioShop) in TBS containing 0.1% Tween-20 (TBS/T, BioShop) prior to incubation with primary antibodies specific for phosphorylated (p)-p38 (#9211), pERK (#9101), p-p65 (#3033), β-actin (#4967) (all from Cell Signaling Technology), TLR2 (clone JM22-41, #MA5-32787) or TLR4 (#48-2300) (both from Invitrogen), at 4 °C overnight. Subsequently, the membranes were washed in TBS/T and incubated with anti-rabbit immunoglobulin secondary antibodies conjugated to horseradish peroxidase (HRP, Dako). Blots were developed using a Clarity Western ECL Substrate (Bio-Rad) and visualized using a ChemiDoc MP Imaging System and the ImageLab software (Bio-Rad).

### TLR2 overexpression

U-251 MG cells were transfected either with an empty pcDNA3.1 vector (control cells), or a vector encoding flag-tagged human TLR2 (the vector was a gift from Ruslan Medzhitov, Addgene plasmid # 13082; http://n2t.net/addgene:13082; RRID:Addgene_13082), using the PEI MAX 40000 reagent (Polysciences), at a reagent to DNA weight ratio of 3:1. For transfection, 1 µg of DNA per well was used in a 12-well plate format. The medium was changed 4 h post-transfection and TLR2 protein expression was assessed by immunoblotting the following day.

### DNA isolation and methylation-specific qPCR

Genomic DNA was isolated using a DNeasy Blood and Tissue Kit (Qiagen) and quantified with a BioPhotometer D30. DNA was digested using the EpiTect Methyl II DNA Restriction Kit (Qiagen) that contains methylation-dependent and methylation-sensitive restriction enzymes. The reaction mixture was incubated overnight at 37 °C, then incubated at 65 °C for 20 min to terminate the reaction. qPCR was performed using the TLR2 promoter CpG-specific primers (EPHS111307-1A; CpG Island 111307) and RT2 SYBR Green qPCR Mastermix (both from Qiagen). The reaction was conducted on a CFX96 Touch™ Real-Time PCR Detection System. The obtained results were analyzed using CFX Manager (Bio-Rad) and the percentage TLR2 promoter methylation was calculated.

### Statistical analyses

Data are presented as the mean + SEM, unless otherwise indicated. For experiments performed on PHGFs or TIGFs, the values of n refer to the number of donors, or number of independent experiments, respectively. The paired t-test, or one-way analysis of variance (ANOVA) followed by the Bonferroni multiple comparison test were used for comparisons between sets of data. P values < 0.05 were considered statistically significant.

## Supplementary Information

Supplementary Figures.

## References

[1] Koliaraki V, Prados A, Armaka M, Kollias G (2020). The mesenchymal context in inflammation, immunity and cancer. Nat. Immunol..

[2] LeBleu VS, Neilson EG (2020). Origin and functional heterogeneity of fibroblasts. FASEB J..

[3] Bautista-Hernández, L. A., Gómez-Olivares, J. L., Buentello-Volante, B. & Bautista-de Lucio, V. M. Fibroblasts: The unknown sentinels eliciting immune responses against microorganisms. *Eur. J. Microbiol. Immunol. (Bp).* **7**, 151–157 (2017). 10.1556/1886.2017.00009 PMC5632742 29034104

[4] Turner JD, Naylor AJ, Buckley C, Filer A, Tak P-P (2018). Fibroblasts and osteoblasts in inflammation and bone damage. Adv. Exp. Med. Biol..

[5] Eke PI (2018). Periodontitis in US Adults: National Health and Nutrition Examination Survey 2009–2014. J. Am. Dent. Assoc..

[6] Hajishengallis G (2014). Immunomicrobial pathogenesis of periodontitis: keystones, pathobionts, and host response. Trends Immunol..

[7] Atkuru S (2021). Cellular ageing of oral fibroblasts differentially modulates extracellular matrix organization. J. Periodontal Res..

[8] Páez J (2020). Uncoupled inflammatory, proliferative, and cytoskeletal responses in senescent human gingival fibroblasts. J. Periodontal Res..

[9] Andrukhov, O., Ertlschweiger, S., Moritz, A., Bantleon, H.-P. & Rausch-Fan, X. Different effects of *P. gingivalis* LPS and *E. coli* LPS on the expression of interleukin-6 in human gingival fibroblasts. *Acta Odontol. Scand.* **72**, 337–45 (2014). 10.3109/00016357.2013.834535 24255960

[10] Lee KM, Choi KH, Ouellette MM (2004). Use of exogenous hTERT to immortalize primary human cells. Cytotechnology.

[11] Meyerson M (1998). Telomerase enzyme activation and human cell immortalization. Toxicol. Lett..

[12] Kassem M, Abdallah BM, Yu Z, Ditzel N, Burns JS (2004). The use of hTERT-immortalized cells in tissue engineering. Cytotechnology.

[13] Kwon Y-D (2014). Cellular viability and genetic expression of human gingival fibroblasts to zirconia with enamel matrix derivative (Emdogain®). J. Adv. Prosthodont..

[14] Illeperuma RP (2012). Immortalized gingival fibroblasts as a cytotoxicity test model for dental materials. J. Mater. Sci. Mater. Med..

[15] Shang L (2018). Multi-species oral biofilm promotes reconstructed human gingiva epithelial barrier function. Sci. Rep..

[16] Shang L (2019). Commensal and pathogenic biofilms alter toll-like receptor signaling in reconstructed human gingiva. Front. Cell. Infect. Microbiol..

[17] Buskermolen JK (2016). Development of a full-thickness human gingiva equivalent constructed from immortalized keratinocytes and fibroblasts. Tissue Eng. Part C. Methods.

[18] Wang, Q., Notay, K., Downey, G. P. & McCulloch, C. A. The leucine-rich repeat region of CARMIL1 regulates IL-1-mediated ERK activation, MMP expression, and collagen degradation. *Cell Rep.* **31**, 107781 (2020). 10.1016/j.celrep.2020.107781 PMC8713033 32610117

[19] Yucel-Lindberg, T. & Båge, T. Inflammatory mediators in the pathogenesis of periodontitis. *Expert Rev. Mol. Med.* **15**, e7 (2013). 10.1017/erm.2013.8 23915822

[20] O’Neill LAJ (2002). Signal transduction pathways activated by the IL-1 receptor/toll-like receptor superfamily. Curr. Top. Microbiol. Immunol..

[21] Davanian H (2012). Signaling pathways involved in the regulation of TNFα-induced toll-like receptor 2 expression in human gingival fibroblasts. Cytokine.

[22] Palm E, Demirel I, Bengtsson T, Khalaf H (2017). The role of toll-like and protease-activated receptors and associated intracellular signaling in *Porphyromonas gingivalis*-infected gingival fibroblasts. APMIS.

[23] Liu J, Wang Y, Ouyang X (2014). Beyond toll-like receptors: *Porphyromonas gingivalis* induces IL-6, IL-8, and VCAM-1 expression through NOD-mediated NF-κB and ERK signaling pathways in periodontal fibroblasts. Inflammation.

[24] de Vries TJ, Andreotta S, Loos BG, Nicu EA (2017). Genes critical for developing periodontitis: Lessons from mouse models. Front. Immunol..

[25] Benakanakere M, Abdolhosseini M, Hosur K, Finoti LS, Kinane DF (2015). TLR2 promoter hypermethylation creates innate immune dysbiosis. J. Dent. Res..

[26] Palm E, Demirel I, Bengtsson T, Khalaf H (2015). The role of toll-like and protease-activated receptors in the expression of cytokines by gingival fibroblasts stimulated with the periodontal pathogen *Porphyromonas gingivalis*. Cytokine.

[27] Burns E, Bachrach G, Shapira L, Nussbaum G (2006). Cutting edge: TLR2 is required for the innate response to *Porphyromonas gingivalis* : Activation leads to bacterial persistence and TLR2 deficiency attenuates induced alveolar bone resorption. J. Immunol..

[28] Liu H, Redline RW, Han YW (2007). Fusobacterium nucleatum induces fetal death in mice via stimulation of TLR4-mediated placental inflammatory response. J. Immunol..

[29] Sun Y, Shu R, Li C-L, Zhang M-Z (2010). Gram-negative periodontal bacteria induce the activation of Toll-like receptors 2 and 4, and cytokine production in human periodontal ligament cells. J. Periodontol..

[30] Beckert B (2019). Immortalized human hTert/KER-CT keratinocytes a model system for research on desmosomal adhesion and pathogenesis of *Pemphigus vulgaris*. Int. J. Mol. Sci..

[31] Shin SH, Park SY, Kim MK, Kim JC, Sung YK (2011). Establishment and characterization of an immortalized human dermal papilla cell line. BMB Rep..

[32] Yin B (2019). Establishment of an immortalized intestinal epithelial cell line from tree shrews by lentivirus-mediated hTERT gene transduction. Cytotechnology.

[33] Kim J (2011). An hTERT-immortalized human urothelial cell line that responds to anti-proliferative factor. Vitro Cell. Dev. Biol. Anim..

[34] Matsumura T (2004). Establishment of an immortalized human-liver endothelial cell line with SV40T and hTERT. Transplantation.

[35] Yang GB, Li XY, Yuan GH, Liu H, Fan MW (2013). Immortalization and characterization of human dental papilla cells with odontoblastic differentiation. Int. Endod. J..

[36] Zhou Y (2015). Noncanonical activation of β-catenin by *Porphyromonas gingivalis*. Infect. Immun..

[37] Moffatt-Jauregui CE (2013). Establishment and characterization of a telomerase immortalized human gingival epithelial cell line. J. Periodontal Res..

[38] Maksylewicz A (2019). BET bromodomain inhibitors suppress inflammatory activation of gingival fibroblasts and epithelial cells from periodontitis patients. Front. Immunol..

[39] Nativel B (2017). *Porphyromonas gingivalis* lipopolysaccharides act exclusively through TLR4 with a resilience between mouse and human. Sci. Rep..

[40] Makkawi H (2017). *Porphyromonas gingivalis* stimulates TLR2-PI3K signaling to escape immune clearance and induce bone resorption independently of MyD88. Front. Cell. Infect. Microbiol..

[41] Spiekstra SW, Breetveld M, Rustemeyer T, Scheper RJ, Gibbs S (2007). Wound-healing factors secreted by epidermal keratinocytes and dermal fibroblasts in skin substitutes. Wound Repair Regen..

[42] Buskermolen JK, Roffel S, Gibbs S (2017). Stimulation of oral fibroblast chemokine receptors identifies CCR3 and CCR4 as potential wound healing targets. J. Cell. Physiol..

[43] Burgess JK (2018). Phenotype and functional features of human telomerase reverse transcriptase immortalized human airway smooth muscle cells from asthmatic and non-asthmatic donors. Sci. Rep..

[44] Futscher BW (2013). Epigenetic changes during cell transformation. Adv. Exp. Med. Biol..

[45] Farwell DG (2000). Genetic and epigenetic changes in human epithelial cells immortalized by telomerase. Am. J. Pathol..

[46] Liu L (2005). A methylation profile of in vitro immortalized human cell lines. Int. J. Oncol..

[47] Gao C (2018). Hypermethylation of PGCP gene is associated with human bronchial epithelial cells immortalization. Gene.

[48] de Faria Amormino, S. A. *et al.* Hypermethylation and low transcription of TLR2 gene in chronic periodontitis. *Hum. Immunol.* **74**, 1231–1236 (2013). 10.1016/j.humimm.2013.04.037 23747679

[49] Kolahi, S. *et al.* Evaluation of DNA methylation status of toll-like receptors 2 and 4 promoters in Behcet’s disease. *J. Gene Med.* **22**, e3234 (2020). 10.1002/jgm.3234 32449979

[50] De Oliveira NFP (2011). TLR2 and TLR4 gene promoter methylation status during chronic periodontitis. J. Clin. Periodontol..

[51] Lagosz KB (2020). HDAC3 Regulates Gingival Fibroblast Inflammatory Responses in Periodontitis. J. Dent. Res..

