# Supplementary material for: hTERT-immortalized gingival fibroblasts respond to cytokines but fail to mimic primary cell responses to Porphyromonas gingivalis
Source: Sci Rep. 2021 May 24;11:10770. doi: 10.1038/s41598-021-90037-5 (PMC8144196; doi:10.1038/s41598-021-90037-5)
Supplement: Supplementary file 1 — Supplementary Figures. [file 41598_2021_90037_MOESM1_ESM.pdf]

hTERT-immortalized gingival fibroblasts respond to cytokines but fail to mimic primary cell responses to *Porphyromonas gingivalis*

Katarzyna B Lagosz-Cwik*, Aleksandra Wielento*, Weronika Lipska, Malgorzata Kantorowicz, Dagmara Darczuk, Tomasz Kaczmarzyk, Susan Gibbs, Jan Potempa and Aleksander M Grabiec

*These authors contributed equally to this work.

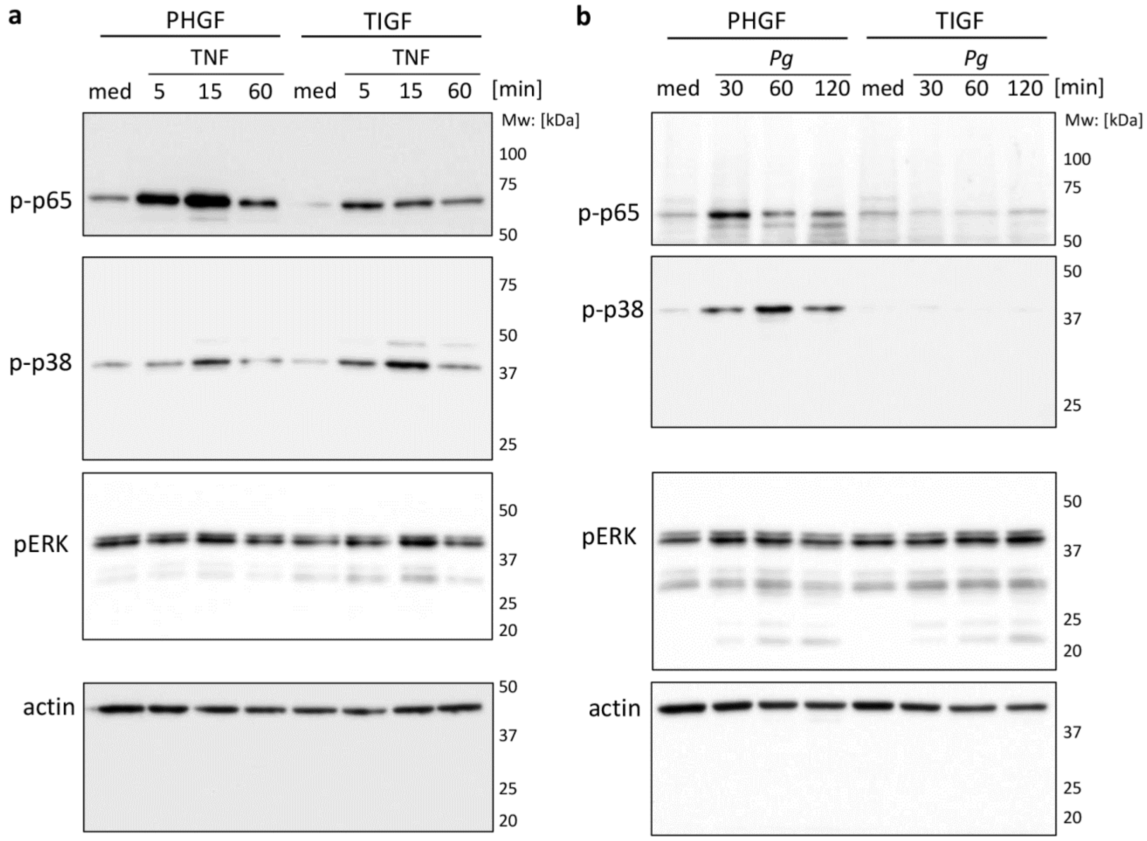

Supplementary Figure S1. Western blot analysis of p65, p38, and ERK phosphorylation in PHGFs and TIGFs (**a**) after stimulation with TNF (10 ng/ml) or (**b**) upon infection with *P. gingivalis* (MOI 50) for the indicated time points (in minutes). Actin was used as loading control. Some membranes were cut at ~50-60 kDa and their top and bottom parts were used to visualize different proteins.

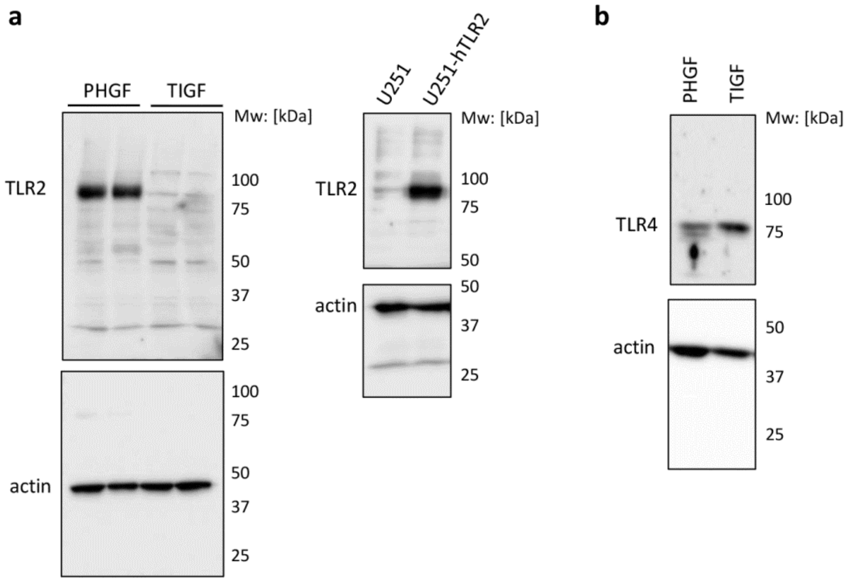

Supplementary Figure S2. Western blot analysis of TLR2 and TLR4 protein expression in total cell lysates of PHGF and TIGF. (**a**) U-251 MG cells overexpressing hTLR2 were used as a positive control and actin was used as a loading control. In the right panel, one membrane cut at 50 kDa was used to visualize TLR2 (top part of the membrane showing molecular weights >50 kDa) and actin (bottom part of the membrane showing molecular weights <50 kDa). (**b**) One membrane cut at ~55 kDa was used to visualize TLR4 (top part of the membrane showing molecular weights >55 kDa) and actin (bottom part of the membrane showing molecular weights <55 kDa).

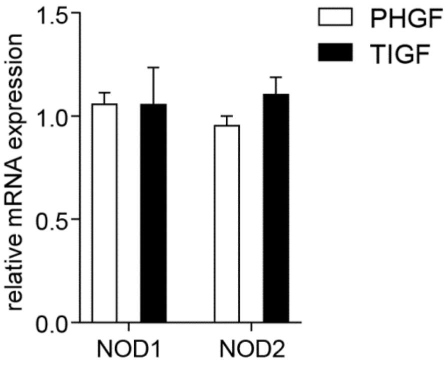

Supplementary Figure S3. PHGFs and TIGF express comparable levels of NOD1 and NOD2. NOD1 and NOD2 expression in PHGFs and TIGFs (n=2) was analyzed by qPCR and shown as mean relative mRNA expression + SEM.
